# In situ transduction of stromal cells and thymocytes upon intrathymic injection of lentiviral vectors

**DOI:** 10.1186/1471-2172-5-18

**Published:** 2004-08-19

**Authors:** Gilles Marodon, David Klatzmann

**Affiliations:** 1 UPMC/CNRS UMR7087, 83 Bd de l'Hôpital, Bât CERVI, 75651 PARIS cedex 13, France

## Abstract

**Background:**

The thymus is the primary site for T-cell development and induction of self-tolerance. Previous approaches towards manipulation of T-cell differentiation have used intrathymic injection of antigens, as proteins, cells or adenoviruses, leading to transient expression of the foreign protein. Lentiviral vectors, due to their unique ability to integrate into the genome of quiescent cells, may be best suited for long-term expression of a transgene in the thymus.

**Results:**

Young adult mice were injected in the thymus with lentiviral vectors expressing eGFP or the hemaglutinin of the Influenza virus under the control of the ubiquitous phospho glycerate kinase promoter. Thymi were examined 5 to 90 days thereafter directly under a UV-light microscope and by flow cytometry. Intrathymic injection of lentiviral vectors predominantly results in infection of stromal cells that could be detected for at least 3 months. Importantly, hemaglutinin expression by thymic stromal cells mediated negative selection of thymocytes expressing the cognate T-cell receptor. In addition and despite the low multiplicity of infection, transduced thymocytes were also detected, even 30 days after injection.

**Conclusions:**

Our results demonstrate that intrathymic delivery of a lentiviral vector is an efficient means for stable expression of a foreign gene in the thymus. This new method of gene delivery may prove useful for induction of tolerance to a specific antigen and for gene therapy of severe combined immunodeficiencies.

## Background

The thymus is a bilobate organ derived from embryonic endoderm and mesoderm differentiation and is located just above the heart (reviewed in [1]). It is the primary organ for maturation of T cells. This process involves the interaction between developing thymocytes and thymic stromal cells. Thymic stromal cells which forms the thymic architecture, have been classified according to their anatomical localization. They encompass a very diverse array of cell types, including cortical and medullar epithelial cells, fibroblasts, macrophages and dendritic cells (reviewed in [2]). Stromal cells control the differentiation of haematopoietic precursors derived from the liver or the bone marrow into T lymphocytes: T-cell differentiation is defined by the acquisition of maturation markers such as CD4, CD8 and the T-cell receptor complex (TCR), which conditions the reactivity of immature thymocytes with thymic stromal cells. The early thymocyte progenitors entering the thymus do not express T-cell-specific molecules, such as CD3, the alpha or beta-chain of the TCR, or the CD4 and CD8 molecules. These CD4^-^CD8^- ^cells, referred to as double-negative (DN) cells, then become CD4^+^CD8^+^, the so-called double-positive (DP) stage, and then progressively acquire TCR molecules. The final maturation of T-cells involves the selective loss of either the CD4 or the CD8 molecules to generate fully mature single-positive (SP) cells with cytotoxic/suppressor or helper/regulator function, respectively.

During this process, the TCR-mediated positive and negative selection of T cells ensures the selection of a diverse TCR repertoire able to react with foreign peptide presented by autologous major histocompatibility complex (MHC) molecules, but tolerant to self-antigens. This property renders the thymus an attractive site for manipulation of T-cell tolerance. To date, results on tolerance induction via direct manipulation of the thymus have been scarce (reviewed in [3]). However, previous studies using intrathymic (IT) injection of pancreatic islet cells [4], soluble antigens [5] or adenoviral vectors [6,7] have shown that induction of tolerance to foreign antigens in non-immunosuppressed animals is feasible. Since the production and maturation of thymocytes may be a life-long process, a major drawback to the utilisation of soluble antigens or adenoviruses is their short-term expression in the thymus [8]. Indeed, modulation of the selection process should stop upon the disappearance of the antigen, which might be a problem for long-term tolerance induction.

Due to their ability to infect resting cells and to stably integrate into the genome, lentiviral vectors represent powerful new tools for long-term expression of a given transgene *in vivo *[9]. Lentiviral vectors have been used successfully *in vivo *to infect hepatocytes and muscle cells [10], antigen-presenting cells [11,12], as well as cells of the central nervous system [13]. We reasoned that lentiviral vectors might be better suited than adenoviral vectors for long-term IT expression of a foreign gene. We therefore investigated the pattern of infection of a ubiquitous lentiviral vector after IT injection. We report herein that thymic stromal cells are massively and persistently infected. Developing thymocytes exhibit a significant but low level of infection. Moreover, we show that IT injection of a lentiviral vector encoding the cognate antigen in TCR-transgenic (Tg) mice leads to negative selection of developing thymocytes.

## Results

### Intrathymic injection of lentiviral vectors results in the efficient and persistent infection of thymic stromal cells

**Figure 1:**
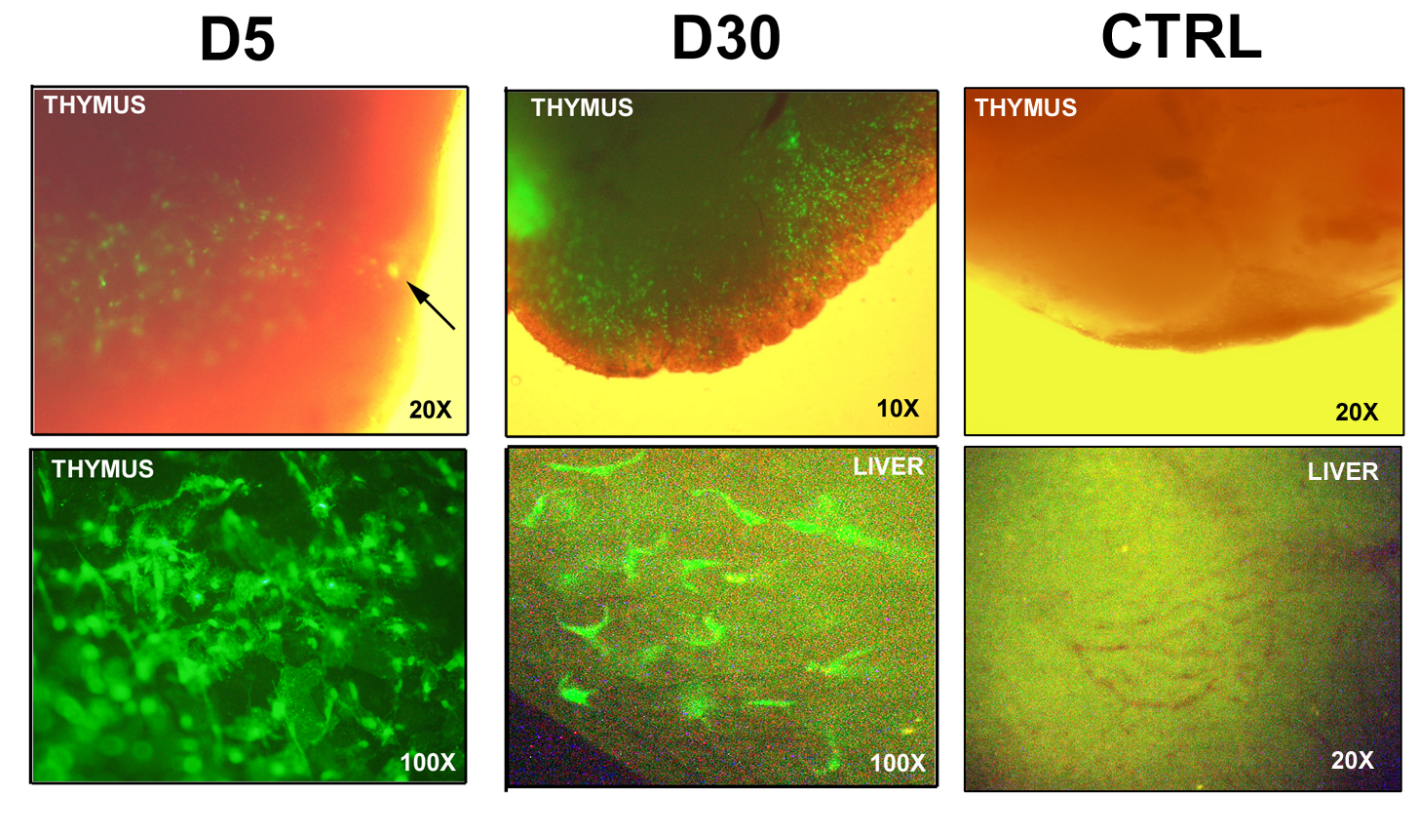
***In vivo *expression of eGFP after intrathymic injection of the LvPGK-GFP vector. **D5: day 5 post-injection localisation of transduced cells around the injection site (arrow) under visible and UV-lights (upper picture). Fibroblast-shaped cells are predominantly transduced (UV-light only) (lower panel). D30: day 30 post-injection expression of eGFP in the thymus (visible + UV-light) (upper panel) and in the liver (UV-light only) (lower panel). CTRL: Thymus (upper panel) and liver (lower panel) pictures from control mice injected IT with PBS examined for background fluorescence under visible and UV-lights. Magnifications are indicated in the lower right corner of each picture.

We used a concentrated viral stock of the LvPGK-GFP vector (2.10^9 ^TU_143B_/ml) to inject between 7 × 10^7 ^to 1.2 × 10^8 ^infectious units in the thymus of normal C57Bl/6 mice. Infected cells could readily be detected at day 5 post-injection by direct examination of the thymi under a UV microscope (figure 1). Of note, transduced cells could still be observed at 1 (figure 1) and 3 months (data not shown) post injection. Most of the transduced cells had a fibroblastic morphology. To monitor a possible passage of the vector through the bloodstream, we checked for the presence of transduced cells in the liver, which is the primary target organ after intravenous injection of lentiviral vectors [14]. We indeed observed numerous eGFP^+ ^cells in the liver (figure 1), suggesting a significant leak into the circulation upon IT injection of up to 30 μl of the vector. Nevertheless, our results demonstrate efficient and persistent infection of thymic stromal cells upon IT injection of a lentiviral vector.

### Induction of negative selection after intrathymic injection of lentiviral vectors

**Figure 2:**
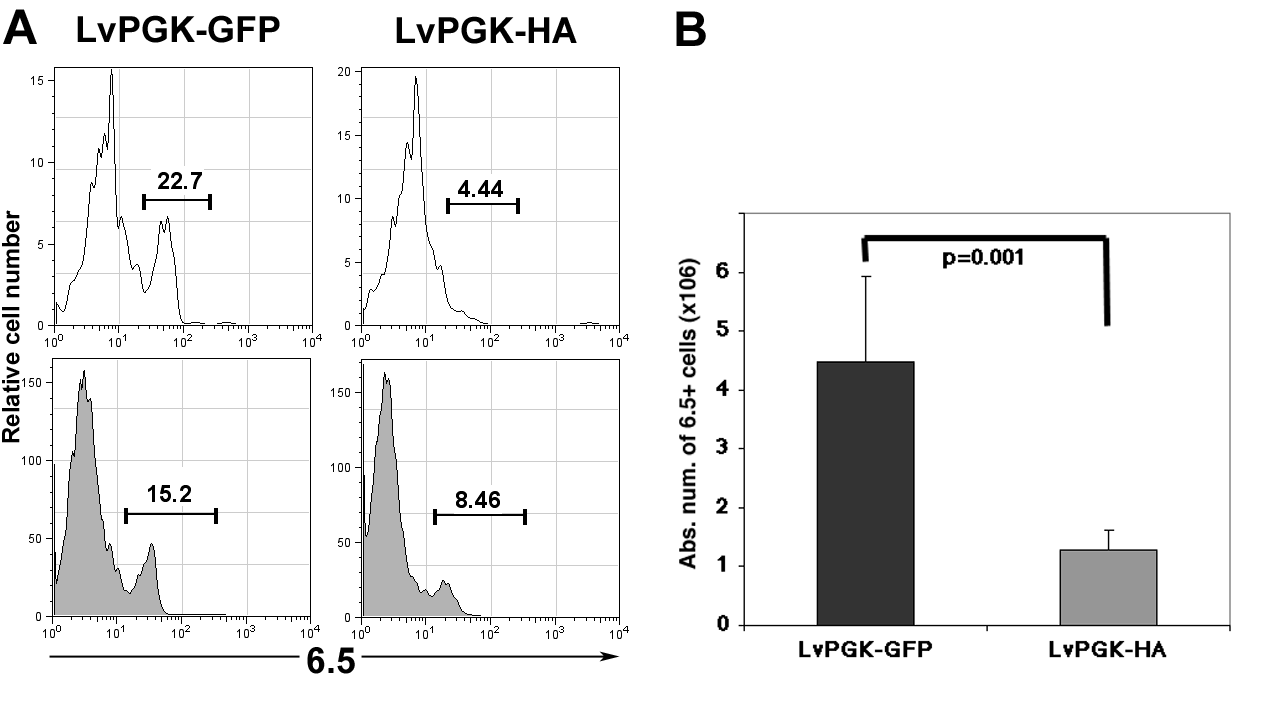
**Negative selection of developing thymocytes (A) **TCR transgenic expression within thymic CD8SP (white) and CD4SP cells (grey) identified by the anti-clonotypic monoclonal antibody 6.5 in SFE-Tg mice six days after IT injection of 40 to 60 ng p24 of the LvPGK-GFP lentiviral vector (n = 2) or of 3.5 to 6 ng p24 of the LvPGK-HA vector (n = 3). Shown are representative profile of two independent experiments. Numbers indicate the frequency of 6.5^+ ^cells **(B) **Absolute counts of HA-specific thymocytes six days after intra thymic injection of LvPGK-GFP or LvPGK-HA lentiviral vectors. These figures were obtained based on the percentages of total 6.5^+ ^thymocytes determined by flow cytometry as shown in (A). Statistical analysis was performed using Student's t-test.

We next wanted to investigate whether thymic stromal cells infected with a lentiviral vector would be able to mediate negative selection of developing thymocytes. We thus injected a lentiviral vector encoding the HA of the Influenza virus, or eGFP as a control, into the thymus of SFE-Tg mice expressing a TCR specific for a HA protein peptide and for which a clonotypic antibody recognizing the transgenic TCR (clone 6.5) is available [15]. Six days after injection, we analyzed the thymocytes by flow cytometry after thymi dilacerations. The frequency of 6.5^+ ^cells within CD4SP and CD8SP thymocytes is shown in figure 2A for a representative experiment. Intrathymic injection of the LvPGK-HA vector resulted in a diminution in the frequencies of 6.5^+ ^cells within CD4SP by a factor of 2 and by a factor of 5 in CD8SP cells with an almost complete disappearance of 6.5^hi ^cells in the latter subset. Of note is that the intensity of TCR transgenic expression was reduced in CD4SP cells (figure 2A). Overall, we observed a 3.5-fold decrease in the total numbers of thymocytes expressing the specific TCR in mice injected with the LvPGK-HA vector compared to the LvPGK-GFP vector (figure 2B). Our results altogether demonstrate that within a week after intra thymic injection of the LvPGK-HA vector, infected thymic stromal cells efficiently mediated negative selection of HA-specific thymocytes.

### Intrathymic injection of lentiviral vectors results in low level infection of immature thymocytes

**Figure 3:**
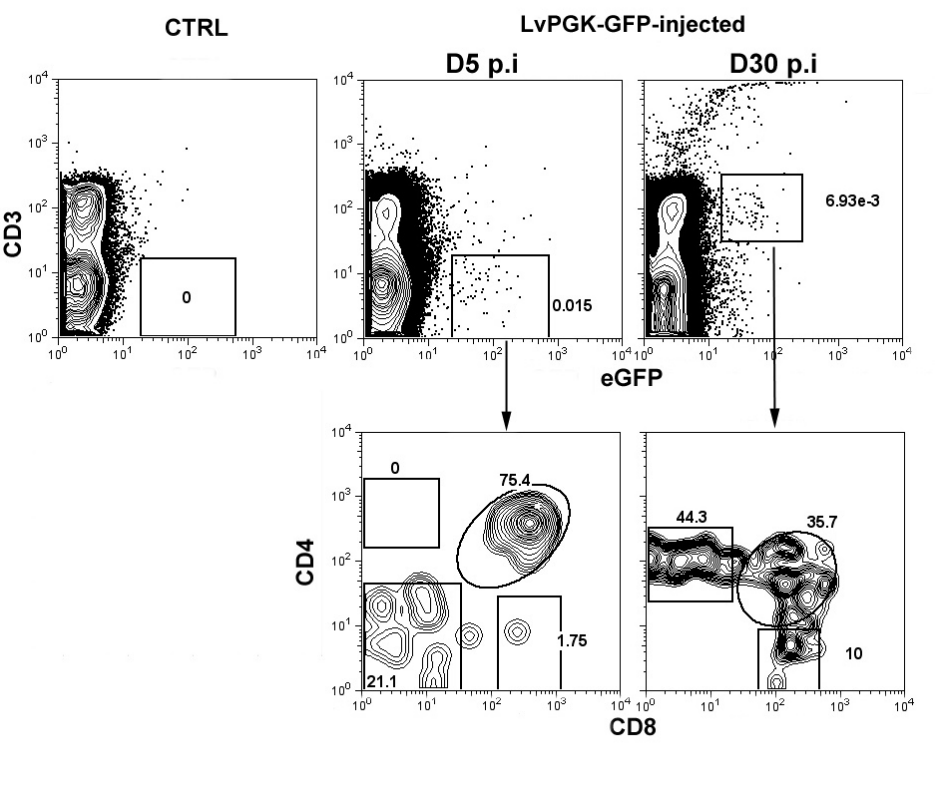
**eGFP expression in developing thymocytes. **Total thymocytes were stained with anti-CD4, anti-CD8 and anti-CD3 monoclonal antibodies. Upper panels: saline-injected control mice (CTRL) and LvPGK-GFP-injected mice at two different time points after injection (D5 and D30) are shown. Lower panels: The profile of CD4/CD8 expression is shown within gated eGFP^+ ^cells.

We next investigated whether developing T-cells would be infected upon IT injection of the lentiviral vector. Five days post-injection of the LvPGK-GFP vector into the thymus of a normal mouse, very few eGFP^+ ^cells could be detected by flow cytometry within the thymocytes obtained from dilacerated thymi (figure 3). Most of these cells were CD3^- ^cells, and belonged to the DN and DP subsets, showing that infected cells were mostly immature. Interestingly, at day 30 post-injection, we observed more mature eGFP^+ ^cells that expressed CD3 and that belonged to the subsets of CD4SP and CD8SP for more than half of them. This result indicates that infection *per se *did not interfere with the normal process of T-cell development. Moreover, we observed a similar repartition of CD4/CD8-expressing cells in non-infected eGFP^- ^cells (data not shown). Collectively, these results show that immature thymocytes can be infected by *in situ *lentiviral infection. However, we were unable to clearly detect infected T-cells in the spleen of injected mice, likely due to their small representation within the pool of mature lymphocytes in the absence of a selective advantage for the transduced thymocytes.

## Discussion

We report herein that IT injection of a lentiviral vector results in the predominant infection of thymic stromal cells, and to a low level infection of thymocyte progenitors. Significant infection of liver cells was also detected. This observation is reminiscent of what was observed by DeMatteo et al. with adenoviral vectors [7]. Together with the fact that liver cells are main targets of IV-injected lentiviral or adenoviral vectors, this suggest that a significant leak into the circulation does occur upon IT injection of viral vectors. Our *in situ *analysis suggests that thymic epithelial cells represent the vast majority of infected cells, and studies are underway to more precisely define the cells targeted by IT lentiviral injection. Whatever the proportion of cortical, medullar epithelial cells, or thymic dendritic cells that are transduced, we show here that this results in an antigen presentation that efficiently mediates negative selection of specific thymocytes. This is not due to the injection of a "crude" preparation of viral supernatant that could have non-specifically affected T cell differentiation. Indeed, we injected 10 times lower amounts of p24 from the LvPGK-HA vector than of the LvPGK-GFP vectors, suggesting that negative selection of HA-specific thymocytes was a direct effect of HA expression by thymic stromal cells. This is further supported by the analysis of the frequencies of 6.5^+ ^thymocytes which shows deletion of 6.5^hi ^cells within CD8SP cells, an MHC class-II restricted population in these TCR-transgenic mice [15]. Down modulation of the transgenic TCR and deletion was observed within CD4SP cells. This is reminiscent of the results obtained recently by Trani et al. which showed that intra thymic delivery of increasing dose of the HA peptide in SFE TCR-Tg mice resulted in the down regulation of the transgenic TCR [16]. Therefore, deletion and/or receptor down regulation may act in concert in the negative selection of HA-specific CD4SP cells in SFE transgenic mice.

A very low infection of developing thymocytes was detected. This is not surprising as (i) the multiplicity of infection (ratio of number of infectious units over number of total cells in the thymus) was estimated to be lower than 0.4 and (ii) lentiviral transduction of murine T cells is far less efficient than of human T cells [17]. Since the actual volume that can be injected in a mouse thymus is however limited, we used the highest MOI achievable with our concentrated vectors. It would be interesting to assess if the use of vectors with higher infectious titres or of repeated injections would lead to a better transduction of thymocytes. It should be stressed though that at day 5 after injection, the infected cells represented immature thymocytes not expressing CD3 molecules. At later time points, infection was detected in more mature thymocytes. We believe that this result may have important implications for *in vivo *gene therapy of severe combined immunodeficiencies (SCID) affecting T-cell development (reviewed in [18]). Indeed, most of these diseases are due to monogenic mutations and concerns immature thymocytes, such as in the T^-^B^+^NK^- ^deficiencies linked to the common cytokine receptor gamma-c [19], or to the ZAP-70 protein tyrosine kinase [20]. Our results open the possibility of correcting these developmental blocks through IT delivery of a lentiviral vector expressing a functional molecule. For this particular application, it would be important to avoid transgene expression in the thymic stroma. The use of our recently described T-cell specific lentiviral vector represent an attractive possibility towards this end [21]. Given the tremendous proliferative potential of T cells and the selective advantage that will be provided by the transgene, even a low number of transduced cells should result in a significant T cell reconstitution. This is best exemplified by a unique case of X-linked severe combined immunodeficiency in which a reverse mutation occurred in a single early T cell precursor. It was determined that at least 1,000 T cell clones with unique T cell receptor-beta sequences were generated from this precursor and that this diversity seems to be stable over time and provides protection from infections in vivo [22]. Furthermore, our preliminary results show that intra thymic delivery of the ZAP-70 gene by mean of a T-cell specific lentiviral vector in ZAP-70-deficient mice results in the restoration of T-cell development (submitted). The presently described *in vivo *approach may represent an alternative to gene therapy protocols using cumbersome haematopoietic stem cell manipulation *ex vivo *prior to their reinfusion *in vivo*.

## Conclusions

Results presented herein may have important implications for the experimental and therapeutic manipulation of the immune system, and notably for tolerance induction and the correction of SCID.

## Methods

### Mice and intrathymic surgery

C57Bl/6 mice were obtained from Charles River/IFFA Credo Laboratories (Les Oncins, France) at 6 weeks of age and were used at 8 to 10 weeks-old. SFE TCR-Tg mice [15] were bred in our own animal facility and were used at 6 to 10 weeks-old. Intrathymic surgery was performed after anesthetic treatment of animals with 40 mg/kg of Pentobarbital (Sanofi-Synthelabo, Gentilly, France). Mid-incision of the lower neck was performed to gain access to the trachea. Incision of the sternum was performed on the first two ribs and gently pulled aside to view the thymus. A single injection of 10 to 30 μl was performed using 0.3 ml Terumo insulin syringes (VWR, Fontenay-sous-bois, France).

### Lentiviral vector construction, production, concentration and quantification

The plasmid encoding the lentiviral vector pRRLsin.PPT.hPGK.GFPpre (LvPGK-GFP) has been described elsewhere [23] (kindly provided by L. Naldini (University of Torino Medical School, Torino, Italy). To construct the plasmid encoding the hemaglutinin (HA) protein of the Influenza virus, BamHI and SalI restriction sites at the 5' and 3' ends, respectively, were added to the cDNA of the HA protein of Influenza virus (H1N1) in the pCIneoHA plasmid (provided by Genethon, Evry, France) by PCR using the Taq polymerase (Invitrogen, Cergy-Pontoise, France). Total PCR products were cloned into the TA vector (Invitrogen), checked for sequence integrity and digested with BamHI/SalI. The plasmid pRRLsin.PPT.hPGK.GFPpre was digested with BamHI/SalI (New England Biolabs, Beverly, Massachussets, USA) to remove eGFP. After ligation, the plasmid pRRLsin.PPT.hPGK.HApre, hereafter referred to as LvPGK-HA, was obtained. To produce lentiviral vectors, a total of 4.10^6 ^293T-cells were co-transfected with the transfer vector, the packaging and the envelope plasmids in 10-cm dishes using the calcium phosphate method as described [21] in DMEM supplemented with serum and antibiotics (Lifetechnologies, Gaithersburg, Maryland, USA). Lentiviral supernatants were collected at 18, 42 and 66 hrs post co-transfection in serum-free DMEM supplemented with antibiotics and L-glutamine, and concentrated by ultrafiltration using either the Ultrafree-15 or the Centricon Plus-80 filter devices according to the manufacturer instructions (Millipore, Bedford, Massachussets, USA). Briefly, supernatants were applied to the filter devices and spun at 2000 g for 20 min. at 20°C. Concentrated supernatants were aliquoted and kept at -80°C until use. Viral stocks of the LvPGK-GFP lentiviral vector were titered on 143B cells as previously described [21]. Viral stocks of the LvPGK-GFP and LvPGK-HA vectors were also quantified using a *gag *p24 ELISA (Zeptometrix, Buffalo, New York, USA).

### Microscopy and images treatment

Whole thymus or liver were excised from injected mice and placed in PBS 1X in a 6-well plate. Pictures of the whole organ were acquired using a DP-11 numeric camera coupled with the CK-40 inverted microscope equipped with a mercury lamp (Olympus France S.A, Rungis, France). Images were processed using Adobe Photoshop (Adobe Systems Inc., San Jose, California, USA).

### Flow cytometry

Thymi were dilacerated between two frosted slides in 1X PBS supplemented with 3% Fetal Calf Serum (Lifetechnologies). Cell suspensions were numerated and 10^6 ^cells were stained with the following monoclonal antibodies (Becton Dickinson Biosciences, le Pont de Claix, France): CD4-APC (allophycocyanin), CD8-CyCr (Cychrome) and either CD3 or pan beta-chain of the TCR-PE (phycoerytrin) or purified anti-clonotypic TCR for the HA peptide SFERFEIFPK presented by MHC class II I-E^d ^(clone 6.5) ([15]) followed by biotinylated anti-rat IgG2b and streptavidin-FITC. Data were collected on a FACScalibur (BD Biosciences) and analysed with FlowJo software (TreeStar, Ashland, Oregon, USA).

## Abbreviations

DN: double negative

DP: double positive

SP: single positive

IT: intra thymic

IV: intra venous

HA: hemaglutinin

MOI: multiplicity of infection

SCID: severe combined immunodeficiencies

## Authors' contributions

GM devised and realised the experiments, analysed the data and wrote the manuscript. DK conceptualised the study and edited the manuscript.
